# Income-related inequalities in health and health services use in Israel

**DOI:** 10.1186/2045-4015-3-37

**Published:** 2014-11-27

**Authors:** Amir Shmueli

**Affiliations:** Department of Health Management and Economics, The Hebrew University–Hadassah School of Public Health, POB 12272, Jerusalem, 91120 Israel

## Abstract

**Background:**

Income-related inequalities in health and in health services use pose a disturbing and challenging issue in health systems, which are based on social health insurance such as Israel.

**Objective:**

To explore income-related inequalities in health and in health services use in Israel in 2009–2010.

**Methods:**

We used the Central Bureau of Statistics file, which linked information on 7,175 households (24,595 persons) from the 2009 Health Survey and the 2010 Incomes Survey. Raw and adjusted concentration curves and indices were calculated for ten chronic conditions (adjusting for age), visits to physicians and hospitalizations (adjusting for health and location).

**Results:**

There is no income-related inequality in asthma and in cancer. The income-related inequality in the remaining eight conditions is ‘pro-poor’, namely, they are more prevalent among poor households. The order of the level of inequality is (from the least unequally distributed): any condition, hypertension, heart diseases, diabetes, depression, respiratory diseases, digestive diseases, and the condition with the highest income-related inequality is activities of daily living (ADL) limitations. The income-related inequality in secondary physicians’ services is ‘pro-rich’. The income-related inequality in primary care is ‘pro- poor’. Hospitalization days are significantly more unequally – ‘pro-poor’ - distributed in the population.

**Discussion:**

International findings are basically similar to the ones found in this paper. Three reasons are believed to have caused these income-related inequalities: the use of preventive services, health behavior and compliance with the doctors’ directions; they might constitute a useful framework for strategizing interventions. The efforts of the Ministry of Health and of the sickness funds launched in 2010 to reduce inequalities should be evaluated by repeating the present analysis with newer data.

## Background

Evidence on income-related inequalities in health and in health services use pose a disturbing and challenging issue in health systems which are based on socialized medicine and social health insurance such as Israel [1–5]. These systems are based on principles of equality, justice and solidarity, adopting the dual equity principle – income-based contributions and health-based consumption of medical care. Evidence that rich persons are healthier or use more health services (adjusted for health state) than poor persons is incompatible with the above principles and causes an alarm. Such inequality is considered ‘avoidable’, as opposed to ‘unavoidable’ health variation caused by age, for example. Clearly, in order to arrive at an estimate of the avoidable income-related health inequality, one must standardize the data for the causes of unavoidable variation.

Past Israeli studies on health and health services use inequalities indicated the existence of significant ethnic, cultural, geographical and educational disparities [6–9]. This paper explores *income*-related inequalities in health and in health services use in Israel, using the concentration methodology, which has been extensively used in other countries (see the above references). This was made possible by a unique data set, which linked the 2009 CBS’s Health Survey with the CBS’s 2010 Incomes Survey.

## Methods

### Data

The data used were taken from a Central Bureau of Statistics file, which linked information on households from two surveys. The first survey, which was conducted in 2009, gathered socio-demographic and health information. The second survey, carried out in 2010, gathered both individual and household information regarding income. Of the 8,713 households (28,968 individuals) who responded to the health survey, 7,175 (24,595) – over 80% - were matched with information from the income survey. The main reason for non-match was the dynamics of households’ formation: individuals change households and the match has no comparative meaning. Naturally, the rate of non-match is higher in the 20–40 age group.

The linked data constitute a unique source of information, including health, use of health services, and income data. Since the relevant income level is that of the household, we chose the household as the unit of analysis.

#### Health

The health survey included information for each individual regarding whether they suffered from each of ten chronic diseases (high blood pressure, heart attack, other heart diseases, stroke, diabetes, asthma, chronic lung disease, chronic disease in the digestive system, cancerous disease and depression or anxieties). We aggregated heart attack, other heart diseases and stroke into a single chronic condition (named heart diseases), leaving us with eight chronic conditions. In addition, we created an indicator if any member of the household suffered from *any* of the above eight conditions. The survey also had a list of activities of daily living (ADL) and information regarding whether the respondent was capable of doing each activity by himself, with assistance or incapable of doing it. We defined an individual as handicapped if she was incapable of doing at least one basic activity without assistance.

For each of the eight chronic conditions, ‘suffering from any condition’, and ADL limitations we defined the household as ‘sick’ if at least one of its members suffered from the condition.

#### Health services use

Data on use of health services comes from the health survey. The household’s yearly number of visits to doctors (family, primary and secondary) is (as is defined by the CBS) the sum of the number of visits of all members during the two weeks preceding the survey multiplied by 26. The household’s yearly number of inpatient days is the sum of yearly inpatient days of all members, where the yearly number of inpatient days for each member is calculated as the number of hospitalizations during the previous year times the length of stay of the last hospitalization. While the length of the last hospitalization does not necessarily represent the mean length of the yearly hospitalizations and thus its use may introduce an error into the calculated number of inpatient days, we expect the error to be small since 70% of those who were hospitalized did so only once (see below in the Results).

#### Income

From the income survey, gross income per standardized household member was used as the indicator of the household’s standard of living (we used the National Insurance Institute’s equivalence scale).

### Statistical strategy

The well-known method of concentration curves and indices (CCs and CIs) was used. First, we created for each chronic condition a raw concentration curve and calculated the raw concentration index. The raw concentration curve plotted the cumulative proportion of households ranked by gross income per standardized member against the cumulative proportion of ‘sick’ households (percentage of ‘sick’ households – in total ‘sick’ households - in the poorest 10%, in the poorest 20% etc.). The raw concentration index equals twice the area between the curve and the diagonal. When each decile of households ranked by income per standardized adult includes a decile of ‘sick’ households, the concentration curve coincide with the diagonal, the concentration index equals zero, and there is no income-related health inequality in the society. The maximal inequality occurs when all the ‘sick’ households are found in the richest or poorest decile of households - the concentration curve is shaped as a triangle (below or above the diagonal respectively) and the concentration index equals 1 or -1. When the concentration curve lies above the diagonal, the concentration index is negative, and this means that the poor are sicker (the inequality is ‘pro-poor’). When the concentration curve lies below the diagonal, the concentration index is positive, and the inequality originates from the fact that the rich are sicker (‘pro-rich’). The raw concentration index and its standard error were calculated using the ‘convenient regression’ method [10]. Since the probability of a household to be ‘sick’ is related to its size, the raw CCs and CIs are adjusted for household size.

While this method is technically simple, intuitive and has been extensively used (see the above mentioned references as well as [11, 12]), it suffers from several conceptual ambiguities. First, as with the univariate version of the CI – the Gini coefficient, its interpretation depends on the choice of the social welfare function and the societal preferences regarding inequality expressed by this choice [13, 14]. For example, the concentration approach focuses on one main variable (income), while adjusting for other determinants. Second, both income and health are components of wellbeing. Income-related inequality in health focuses thus on a single dimension of inequality in wellbeing, using the correlation between the two. The critics of the concentration approach advocate that it should be complemented with the use of multidimensional inequality measures or with the measurement of inequality in some overall measure of wellbeing.

The raw concentration analysis of health services use is similar: The cumulative percent of the households’ yearly use of health services – out of total use - is plotted against the cumulative proportion of households ranked by gross income per standardized member. The raw concentration index is calculated as above. Since the household use of medical care is related to its size, the raw CCs and CIs are adjusted for the size of the households.

Since there are several additional factors which determine the health of the individual and her use of health services beyond income, the raw concentration curve and index were adjusted in order to reflect just the avoidable income-related health inequality. We focused on a major determinant of health – and of unavoidable health inequality – age. We used the age of the household head to represent the household’s age. In order to account for the effect of the age of the household on its level of morbidity, we calculated the standardized concentration curve and index (they are adjusted for the household size as well). The standardization consists of eliminating the effect of age on health and on income using an auxiliary regression, which also yields the statistical test of the CIs [12].

The standardized concentration curve and index for the use of health services are adjusted for two main determinants of the demand for and the supply of medical care: health state (the number of chronic conditions in the household) and peripheral status of the household’s residence (periphery, intermediate and center, defined by the CBS). They are adjusted for the household size as well.

## Results

### Concentration analysis for health state

Table 1 presents the proportions of ‘sick’ households in each of the nine conditions. The most prevalent condition is hypertension, where over a third of households include at least one person with hypertension. Persons with heart diseases are found in 17% of the households, and diabetes – in 16%. In 11% of the households, at least one member was diagnosed as suffering from asthma, and in 11% - from ADL limitations. In 9% of the households at least one member was diagnosed as suffering from digestive disease, and in 8% - from depression. The least prevalent conditions are cancer (6%) and respiratory diseases (5%). More than half of the households include a member who suffers from any of the eight conditions.

**Table 1:** **The mean number of persons with chronic conditions per household**

Any chronic condition	0.551
Hypertension	0.338
Heart diseases (inc. stroke)	0.167
Diabetes	0.162
Asthma	0.111
Respiratory disease	0.054
Digestion disease	0.089
Cancer	0.055
depression	0.075
ADL	0.109

**Figure 1:**
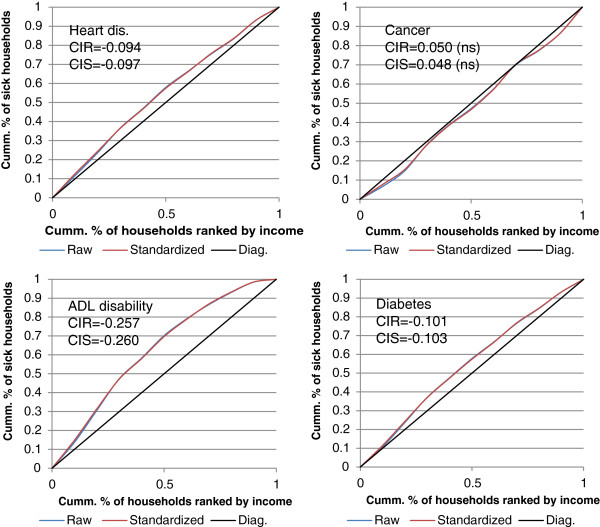
Concentration curves and indices for selected chronic conditions*. *CIR = Raw concentration index; CIS = Standardized concentration index.

Table 2 presents the raw and standardized CIs for the ten conditions investigated. The two CIs are very close for all conditions. Figure 1 shows the concentration curves for heart diseases, diabetes, cancer and ADL limitations.

**Table 2:** Raw and adjusted concentration indices*

	Raw					Adjusted				
	Coef.	Std. Err.	t	95% Conf. interval		Coef.	Std. Err.	t	95% Conf. interval	
Hypertension	**-0.023**	0.010	-2.400	-0.042	-0.004	**-0.025**	0.009	-2.940	-0.042	-0.008
Heart diseases	**-0.094**	0.015	-6.160	-0.124	-0.064	**-0.097**	0.015	-6.610	-0.126	-0.068
Diabetes	**-0.101**	0.015	-6.520	-0.131	-0.071	**-0.103**	0.015	-6.820	-0.133	-0.073
Asthma	0.004	0.020	0.180	-0.036	0.043	0.003	0.020	0.170	-0.036	0.043
Respiratory diseases	**-0.148**	0.026	-5.750	-0.199	-0.098	**-0.150**	0.026	-5.830	-0.201	-0.100
Digestive diseases	**-0.172**	0.021	-8.120	-0.214	-0.130	**-0.174**	0.021	-8.250	-0.215	-0.133
Cancer	0.050	0.027	1.850	-0.003	0.104	0.048	0.027	1.770	-0.005	0.100
Depression	**-0.111**	0.023	-4.860	-0.156	-0.066	**-0.112**	0.023	-4.940	-0.157	-0.068
Any chronic condition	**-0.014**	0.007	-2.18	-0.027	-0.001	**-0.016**	0.006	-2.62	-0.028	-0.004
ADL limitations	**-0.257**	0.018	-14.420	-0.292	-0.222	**-0.260**	0.017	-14.960	-0.294	-0.226

*Raw CIs are adjusted for household size. Standardized CIs are adjusted for household size and age of the household head.

Bold = significant at 0.05.

There is no income-related inequality – neither using raw data nor adjusting for age - in asthma and in cancer. The income-related inequality in the remaining eight conditions and for ‘any condition’ is pro-poor (meaning that the share of poor households in total ‘sick’ households exceeds their share in the population). The prevalence of any condition is the least unequally prevalent condition (the standardized CI is -0.016). Hypertension comes second (CI = -0.025), followed by heart diseases (CI = -0.097), diabetes (standardized CI = -0.103), depression (CI = -0.112), respiratory diseases (-0.150), digestive diseases (-0.174), and the condition with the highest income-related inequality is ADL limitations (-0.260).

### Concentration analysis for health services use

Table 3 presents the mean yearly number of visits and inpatient days per household. The mean number of visits to primary physicians (which includes family physicians, gynecologists and pediatricians) is 17.6, to family physicians – 13.1, and to secondary physicians – 5.5. The mean yearly number of inpatient days per household was 2.2 (since, as was described above, the yearly number of inpatient care was calculated with a potential error, we compared the total number of inpatients days according to our calculation - 5 million = 2.3 million households times 2.2 – to the total number of inpatient days reported by the MOH (Inpatient Institutions and Daycare Units in Israel – 2012). This was 5.2 million.

**Table 3:** **The mean number of yearly visits and inpatient days per household**

Family physicians	13.100
Primary physicians	17.633
Secondary physicians	5.519
Inpatient days	2.178

**Figure 2:**
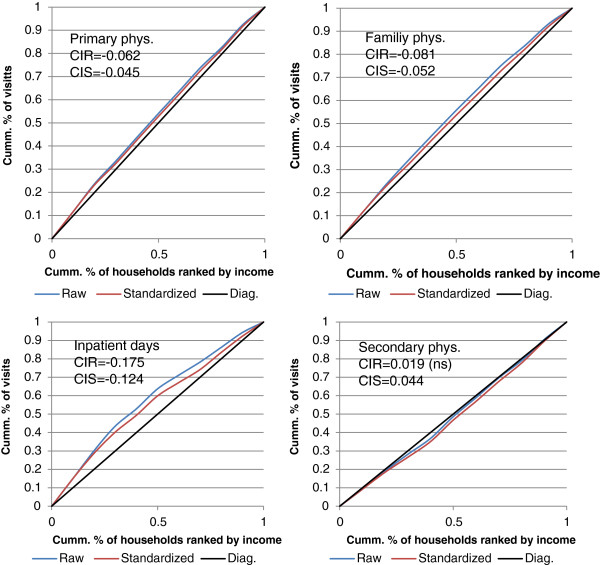
**Concentration curves and indices for the use of health services.** *CIR = Raw concentration index; CIS = Standardized concentration index.

Table 4 shows the raw and standardized CIs for the four health services considered. The two sets are clearly different, with the standardized CIs being larger than the raw ones. Figure 2 shows the concentration curves.

**Table 4:** Raw and standardized health services use concentration indices*

	Raw					Standardized				
	Coef.	Std. Err.	t-value	95% Conf. interval		Coef.	Std. Err.	t-value	95% Conf. interval	
Hospitalizations	**-0.175**	0.030	-5.870	-0.234	-0.117	**-0.124**	0.030	-4.170	-0.183	-0.066
Primary physicians	**-0.062**	0.012	-5.110	-0.086	-0.038	**-0.045**	0.012	-3.770	-0.068	-0.022
Family physicians	**-0.081**	0.014	-5.790	-0.108	-0.053	**-0.052**	0.013	-3.880	-0.079	-0.026
Secondary physicians	0.019	0.020	0.950	-0.020	0.059	**0.044**	0.020	2.220	0.005	0.084

*Raw CIs are adjusted for household size. Standardized CIs are adjusted for household size, number of chronic conditions in the household and its geographical location.

Bold = significant at 0.05.

Secondary physicians’ services are equally distributed – with respect to income - in the population using raw data, but become pro-rich when standardized further for health and location (CI = 0.044). This means that the share of yearly visits to specialists made by rich households is higher than their share in the population. The income-related inequality in primary care is pro-poor (CI = -0.045), namely, the share of visits to family physicians, gynecologists, and pediatricians made by the poor exceeds their share in the population, adjusting for health state and location. Visits to family physicians are somewhat less equally (but still pro-poor, CI = -0.052) distributed. Hospitalization days are by far the most unequally – pro-poor - distributed in the population. The raw CI is -0.175. Controlling for health state and peripheral status, the inequality drops, and the standardized CI is -0.124.

## Discussion

The results indicate that there were significant income-related inequalities in health and in health services use in 2010 Israel. Apart from asthma and cancer, which have no income-related inequality in their prevalence, poor households are sicker (after standardizing for age and household size) in each of the remaining eight conditions. Listed from the least unequally distributed to the most, these conditions are: hypertension, heart diseases, diabetes, depression, respiratory diseases, digestive diseases and ADL limitations. Even when considering ‘any condition’ – where half of the households are sick – the income-related inequality is significantly pro-poor.

The interpretation of the findings rests on the assumption that income affects health. However, the correlation of health and income could also be due to an affect of health on earning power and income. While the chronic conditions discussed in the paper do not generally limit earning capacity, ADL limitations might do. Consequently, the relatively large income-related inequality in ADL limitations might result from the two-way causation.

After standardizing for household size, health needs (which affect the demand for services) and peripheral location (which can affect the supply of services), the use of secondary physicians’ services is distributed in a pro-rich way, while the distribution of primary and inpatient care is unequally distributed with significant pro-poor tendency. Inpatient care is distributed in a more pro-poor manner than primary care. Standardization for needs and supply decreases the observed (raw) pro-poor inequality, since poor households are likely to be sicker and to reside in the periphery where medical care is less available.

Essentially similar results have been reported from other countries in the literature. Regarding income-related inequality in health, van Doorslaer and Koolman [15], and van Doorslaer and Masseria (2004) based on data from a survey taken in 1996 in 13 EU countries, used the individual self-assessed health given in 5 categories. They found that the concentration index (CI) range from 0.0034 (the Netherlands) to 0.0218 (Portugal), namely, rich persons tend to report better health, but the level of inequality is relatively small. In an earlier Israeli study, Shmueli and Gross [16] found similarly that the standardized CI for income-related inequality in self-reported health in 1999 was 0.03. Kakwani, Wagstaff, and van Doorslaer [10], based on data from a Dutch survey carried out in 1980–1981, used the prevalence of (any) chronic diseases as the health indicator, and found that poor persons are sicker, but the level of inequality is small: the raw CI is -0.0404 and the standardized CI is -0.0098. This standardized CI is rather close to the one we found in the present study (-0.016). De Looper and Lafortune [4] conclude, after analyzing a vast array of health indicators for the OECD countries, that “In each case, people in lower socioeconomic groups tend to have a higher rate of disease, disability and death’.

**Figure 3:**
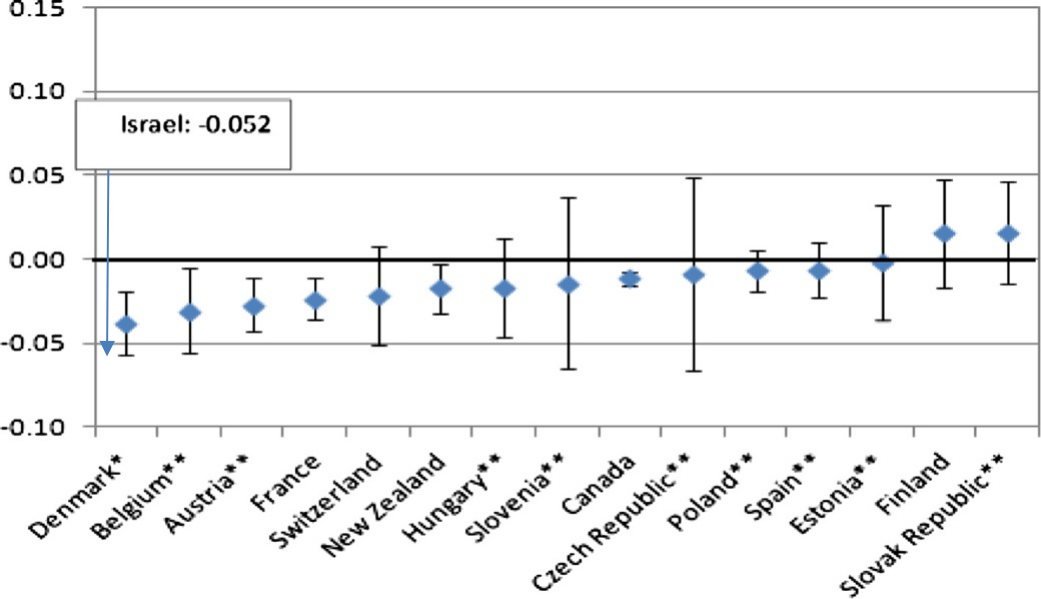
**CI for GP visits in the past 12 months, adjusted for need, 2009 (or latest year).** Source: For Israel – the present study. For the other OECD countries – Devaux and de Looper [4].

Devaux and de Looper [5] provide recent comparative CIs of income-related inequality in the use of GPs and specialists (secondary) care. The comparison is not totally valid since they focus on individuals while we focused on households, and the standardization was somewhat different. Figure 3 presents their findings on standardized CIs of GPs visits in 15 OECD countries. Our estimate of the corresponding Israeli index is -0.052, which is the lowest among the 15 countries. In other words, after adjusting for health needs, the Israeli poor enjoy, relatively to the rich, more family doctors’ services than in other countries. One of the reasons is probably the zero (or very low)-copayment demanded for family doctors visits in Israel. Since poor persons are more sensitive to lower prices than rich persons, their demand for family physician care is expected to be higher, adjusting for health needs.

**Figure 4:**
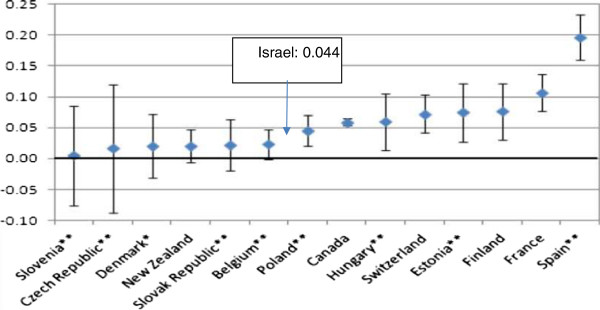
**CI for specialist visits in the past 12 months, adjusted for need, 2009 (or latest year).** Source: For Israel – the present study. For the other OECD countries – Devaux and de Looper [4].

Figure 4 presents the results for specialists’ visits. In all countries, the income-related inequality is pro-rich. Israel – with an index of 0.044 – is located at the center of the range, with pro-rich inequality lower than that in Canada, Switzerland and France. One of the explanation for that picture is the relatively low copayment (about 5 euros for a quarter) for specialist care in Israel.

## Conclusions

The findings that poor households are more intensive users of primary and inpatient care leads to the conclusion that the Israeli publicly-financed health system functions equitably – poor persons who are (more severely) sicker than rich persons use more health services. The disturbing question is why, in a publicly financed health system, are poor persons sicker and suffer from higher severity of the conditions than rich persons? Three reasons are believed to contribute to these income-related inequalities: the use of preventive services, health behavior and compliance with the doctors’ directions. Poor people use less preventive services, conduct worse health behavior and more often do not fully understand and comply with the doctors’ directions ( [17, 18]). Interventions aimed at reducing income-related inequalities should be based on these channels for change.

The data used is dated to 2010. In that year, the Ministry of Health launched a coordinated campaign to reduce health inequalities. It included, among other measures, the addition of peripheral status to the risk-adjustment scheme, which determines the allocation of the health budget among the sickness funds; the implementation of payments to the sickness funds conditional on their efforts to reduce inequalities; and the change in the relative payments to physicians and increasing inpatient facilities in the periphery. Recent MOH reports [19, 20] summarize the many recommendations offered to tackle health inequalities in Israel and the specific policy initiatives implemented during 2010–2013. No effort has been done thus far to evaluate the actual health inequalities in the present. The present paper offers a baseline set of measures of income-related health inequalities to which future similar research can be compared.

## Authors’ information

Amir Shmueli is a professor of health economics at The Hebrew University–Hadassah School of Public Health. His current research interests include inequalities in health and solidarity in healthcare systems.
